# Effectiveness of virtual reality therapy in the treatment of anxiety disorders in adolescents and adults: a systematic review and meta-analysis of randomized controlled trials

**DOI:** 10.3389/fpsyt.2025.1553290

**Published:** 2025-02-27

**Authors:** Weisi Zeng, Jialan Xu, Jiayan Yu, Xin Chu

**Affiliations:** ^1^ School of Nursing, Chengdu University of Traditional Chinese Medicine, Chengdu, Sichuan, China; ^2^ Hospital of Chengdu University of Traditional Chinese Medicine, Chengdu, Sichuan, China

**Keywords:** virtual reality therapy, virtual reality exposure therapy, adolescent, adult, anxiety, anxiety disorder

## Abstract

**Background:**

To evaluate the effect of virtual reality therapy on anxiety disorders in adolescents and adults by Meta-analysis

**Methods:**

A comprehensive literature search was conducted by PubMed, Web of Science, Embase, CINAHL, Scopus, Cochrane (from inception to November 2024). Two researchers independently performed literature screening, quality evaluation and data extraction, and Stata16.0 and Review Man 5.4 software were used for Meta-analysis.

**Results:**

A total of 33 studies involving 3182 adolescents and adults with anxiety disorders were included. The results of Meta-analysis showed that compared with conventional interventions, virtual reality therapy significantly improved the symptoms and level of anxiety in patients with anxiety disorder [SMD = -0.95, 95%CI (-1.22,-0.69), Z = 7.05, P < 0.00001].

**Conclusion:**

The current findings suggest that VR therapy interventions have a positive effect on improving the anxiety state of patients with anxiety disorders. The aforementioned research findings must be confirmed by more high-caliber studies due to the number and quality limitations of the contained literature.

**Systematic review registration:**

https://www.crd.york.ac.uk/prospero/display, identifier CRD42024574772.

## 1 Introduction

Anxiety disorders can be classified as either substance/drug-induced or non-substance/drug-induced. Panic disorder, agoraphobia, separation anxiety disorder, social anxiety disorder, specific phobia, obsessive-compulsive disorder, generalized anxiety disorder, acute stress disorder, post-traumatic stress disorder, illness anxiety disorder, adjustment disorder with anxiety, and anxiety disorder resulting from physical diseases are among the categories of anxiety disorders that are not caused by substances (1). The two most prevalent forms of anxiety disorders are panic disorder and generalized anxiety disorder (2). Generalized anxiety disorder, another name for chronic anxiety disorder, is typified by persistent, excessive, and uncontrollable worrying. It can also be accompanied by a number of physical and psychological symptoms. Anxiety disorders are associated with high comorbidity rates, with 68% of GAD patients report having at least one other mental condition (2, 3). Recurrent, unplanned panic attacks are a hallmark of panic disorder, sometimes referred to as acute anxiety attacks (4, 5). Patients with panic disorder and generalized anxiety disorder continue to worry excessively and be overly vigilant, which is accompanied by considerable functional impairment. In extreme situations, it may cause severe autonomic symptoms, which lowers the quality of life for anxiety disorder sufferers (6). The intricate interaction of biological factors, environmental effects, and psychological mechanisms leads to anxiety (7). According to certain studies, state anxiety and trait anxiety are closely associated with patients who suffer from generalized anxiety disorder (8). The prevalence of anxiety disorders is currently estimated to be between 0.9% and 28.3% (9).

During this critical time in their physical and mental development, adolescents are particularly vulnerable to anxiety, sadness, and other negative feelings brought on by the demands of their studies, families, interpersonal relationship, and other aspects. Teenagers in a fast-paced world face stress from family dynamics, lifestyle changes, and academic demands, all of which have a negative impact on their mental health development (10). The prevalence of psychological issues among teenagers today is rising, including social phobia, depression, and anxiety. According to a 2015 meta-analysis of 41 research conducted in 27 different countries, between 11% and 16% of children and adolescents globally experience at least one mental health problem (11). Adolescents and adults with anxiety disorders have more serious social and educational impairments, and people with anxiety are at higher risk of smoking, substance abuse, obesity, and suicide. It is evident that anxiety and its associated problems significantly affect adults’ and teenagers’ lives and careers.

A variety of therapies, including as pharmaceutical, physical, and psychological ones, can considerably lessen the symptoms of anxiety. It has been demonstrated that mindfulness-based stress therapy (MBSR) and mindfulness-based cognitive therapy (MBCT), two therapies that have been adapted from mindfulness interventions, are successful in lowering anxiety (12–15). There is proof that fitness training and music therapy both have steadily improving impacts on anxiety (16, 17). Preclinical trials and investigations involving human subjects have also demonstrated the efficacy of repetitive transcranial magnetic stimulation’s anxiolytic effects (18). In addition, a meta-analysis showed (19) that pharmacotherapy, cognitive behavioral therapy, and a combination of the two treatment modalities all resulted in significant improvements before and after treatment, and that pharmacotherapy was equally effective as psychotherapy. Only a minority percentage of people receive Cognitive Behavior Therapy (CBT), despite the fact that it is the best first line of treatment for anxiety symptoms (20), and that numerous studies have shown its efficacy in treating anxiety-related diseases (21–24). Alternative methods of treating anxiety disorders are required in light of the acceptance of behavioral cognitive therapy (25).

Virtual reality (VR) is a brand-new technology developed since the 20th century. This computer technology synthetically simulates the environment and stimulates various senses through an immersive experience, allowing users to experience realistic, immersive feelings in a three-dimensional simulation (26) and helps people better regulate their emotions by diverting their attention from pain and anxiety (27). Immersion, interactivity, and imagination are the fundamental features of virtual reality technology. The most popular type of virtual reality is immersive virtual reality, which creates a multi-dimensional and multi-sensory virtual world. Through the use of virtual reality headset display devices and headphones, patients are separated from the outside world and sound, allowing their entire body and mind to be submerged in the dynamic visual virtual environment (28). Researches have demonstrated that the use of immersive head-mounted display displays can successfully divert patients’ focus by placing them in realistic scene settings and have a favorable intervention effect on the control of anxiety during surgery (29–33). Researchers in the field of psychology have also proposed virtual reality therapy (VRT). North et al. (34) defined VRT as “an effective treatment method by exposing clients to stimuli similar to those experienced in the real world through computer-generated virtual reality”. The use of virtual reality therapy has expanded beyond exposure therapy (35), to include behavioral cognitive therapy (36), art therapy (37), and sandplay therapy (38) due to the ongoing advancements in virtual reality technology. Simultaneously, VR technology is more widely accepted, has greater image quality, and is less expensive than standard treatment (39). According to one study, virtual reality exposure therapy (VRET) was favored by 76% of participants over conventional exposure therapy (40).

Virtual reality technology is currently being used progressively in the field of mental health, primarily for the clinical assessment and management of mental illnesses (41). It involves evaluating social functioning, behavioral ability, cognitive function, and symptoms (42–44). Patients can also receive mindfulness therapy, virtual reality exposure therapy, and cognitive rehabilitation (45, 46). Virtual reality therapy has emerged as a significant adjunctive treatment modality in the healthcare industry, with applications in pain management (47, 48) and the treatment of post-traumatic stress disorder (49). However, there is a dearth of scientifically supported data regarding the impact of virtual reality therapy interventions on patients suffering from anxiety disorders. This study uses meta-analysis to systematically evaluate the effect of virtual reality therapy in the treatment of patients with anxiety disorder, so as to provide evidence-based basis for researchers and clinicians.

## 2 Methods

This meta-analysis was completed in accordance with the Preferred Reporting Items for Systematic Reviews and Meta-Analyses statement (50) and was registered with PROSPERO (Registration NO: CRD42024574772).

### 2.1 Search strategy

The researches on the application of artificial intelligence in adolescent mental illness were searched using medical specialty databases of PubMed, Web of Science, Embase, CINAHL, Scopus, Cochrane. The retrieval time was from the establishment of the database to September 2024. In addition, the references of the included literature were traced back to supplement the acquisition of relevant literature. Retrieve take subject and words combination of freedom. Search terms include: anxiety, angst, nervousness, anxiousness, anxiety disorder, adolescent, youth, VR, virtual reality immersion therapy, virtual reality therapy, virtual reality, etc. (The detailed search terms are provided in **Supplementary Table S1**).

### 2.2 Eligibility criteria

Included studies were randomized controlled trials (RCTs) of adolescents and adults with a diagnosis of anxiety disorder, with no restrictions on population gender, course of illness, or ethnicity. The outcome measures included any clinically validated rating scale assessing change in symptoms or scores of anxiety disorders at post-treatment and follow-up, with a well-defined assessment methodology and measurements, such as the Hamilton Anxiety Scale(HAMA), the Self—Rating the Anxiety Scale(SAS), the Beck Anxiety Inventory(BAI), and others. The experimental group received a virtual reality-based intervention, while the control group received at least one non-virtual reality-based intervention, including cognitive behavior therapy(CBT), mindfulness therapy(MT), treatment as usual (TAU) or other interventions. Only articles in English were included.

### 2.3 Data extraction

All retrieved literature was imported into Endnote 20 software, duplicates were removed, and initial screening was performed by 2 researchers reading the titles and abstracts of the remaining literature according to the inclusion and exclusion criteria. After the initial screening the full text was read again to determine the final inclusion of literature. All relevant data were independently screened, extracted and cross-checked by two researchers (ZWS XJL), and each study included basic information about the study, participant characteristics, intervention, control group and data on outcome indicators.

All data were reviewed by a third researcher(YJY), and uncertainties or inconsistencies were resolved by discussion. For the literature lacking information, try to contact the original authors to supplement it.

### 2.4 Quality appraisal

The Cochrane Risk of Bias Assessment Tool was utilized to evaluate the risk and quality of the methodology. A total of six aspects of the included literature were used to evaluate the methodological quality and risk of bias: selection bias, implementation bias, measurement bias, follow-up bias, reporting bias and other biases. According to the evaluation criteria, a judgment was made for each study: “High” indicated that the methodology of the included study was incorrect and its quality was high. “Low” indicates that the methodology of the study was correct and the quality of the study was at low risk, while “Unclear” indicates that the current data are not sufficient to judge the correctness and quality of the methodology. The evaluation grade was A when the included literature fully satisfied the low risk of bias, indicating excellent literature quality; B when it partially satisfied the low risk of bias, indicating fair literature quality; and C when it fully failed to meet the low risk of bias, indicating poor literature quality.

Two researchers independently reviewed the screening procedure and quality evaluation to limit the subjectivity of the researchers and to ensure the credibility of the screening process. If there was a disagreement, it was settled through discussion, and a third party had to be involved in the decision-making process.

### 2.5 Statistical analysis

Review Man 5.4 software was used for data analysis. The 95% Confidence Interval (CI) for continuous variables was presented as either the Weighted Mean Difference (WMD) or the Standardized Mean Difference (SMD). For outcome data, the standardized mean difference (SMD) and 95% confidence interval (CI) were employed due to the nonuniformity of the scales utilized.

The Cochrane Q test was employed to gauge the degree of heterogeneity among the results of each study. The outcome data were homogeneous when P>0.1 and I2 ≤ 50%, and the fixed effect model was chosen to determine the combined effect size. When P ≤ 0.1 and I2>50%, the heterogeneity was considerable, and the reasons for heterogeneity could be found through baseline data, intervention measures, intervention time and other aspects. Descriptive analysis or subgroup analysis of effect sizes can be used to incorporate the heterogeneity caused by the aforementioned factors. The random-effects model was selected for investigation in the event that the source of heterogeneity could not be identified.

### 2.6 Publication bias and additional analysis

Stata16.0 software was used for sensitivity analysis. Egger’s test and funnel plot were used to assess publication bias.

## 3 Results

### 3.1 Study selection

**Figure 1:**
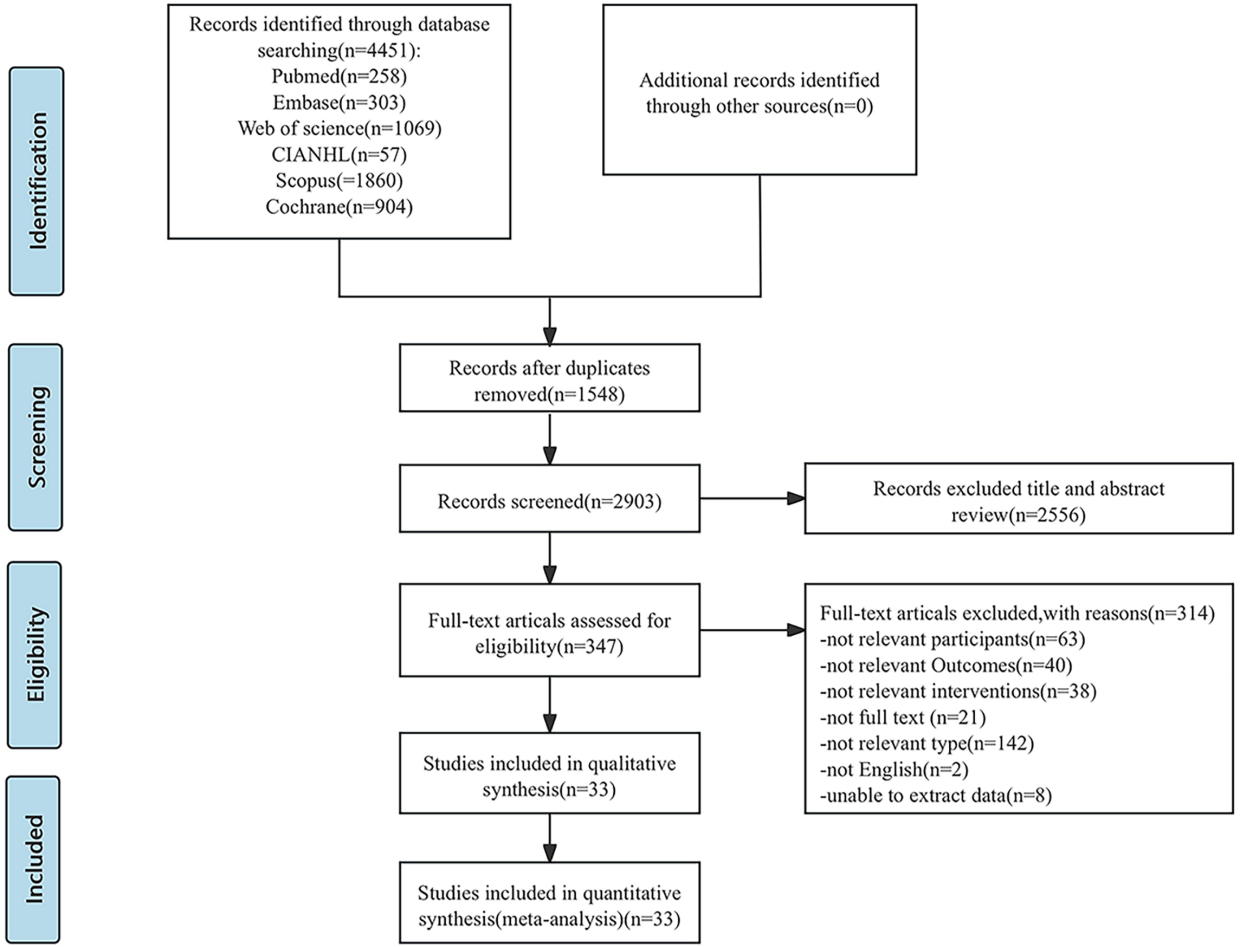
PRISMA flowchart: overview of in- and exclusion process. n, number.

A total of 4451 relevant studies were retrieved. The software Endnote 20 was used to import all of the recovered literature. After deleting the duplicate research, a total of 2903 relevant studies were found. After reviewing the study titles and abstracts, 2556 articles were rejected based on the inclusion and exclusion criteria of this investigation. After re-reading the full text of the literature that might meet the inclusion criteria, 314 studies were excluded, and 33 studies were finally included. Included in the study. **Figure 1** depicts the precise procedure and outcomes of the literature screening

### 3.2 Characteristics and quality of included studies

**Figure 2:**
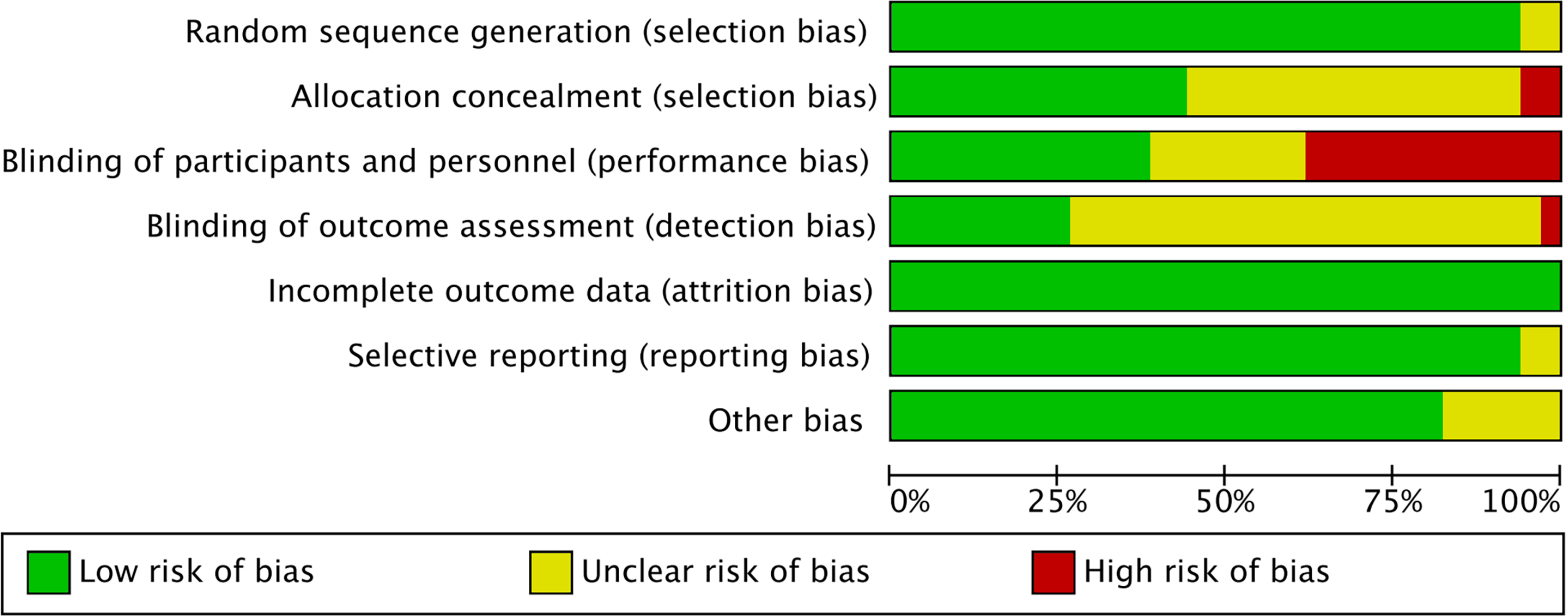
Risk of bias graph.

A total of 33 articles (51–83) were included, published from 2019 to 2024, involving 3182 subjects from 18 countries, including the United States, China, and Iran etc. Out of the 33 included papers, 6 employ three-arm controlled studies (56, 57, 62, 71, 78, 80) and 27 employ two-arm controlled studies. Only two intervention groups were chosen for comparison since unit risk bias did not need to be taken into account with the three-arm controlled trial since each group was independent. One study had a low risk of bias, sixteen had an unclear risk of bias, and sixteen had a high risk of bias out of the 33 included studies. There is a significant risk of implementation bias and measurement bias because most studies cannot be completely double-blind due to the requirement for virtual reality equipment in VR therapy. At the same time, the risk of bias in the selection of the included literature is also high, which could be brought on by inconsistent investigation methods and a lack of representative samples. The basic characteristics of the included literature are shown in **Table 1**. The results of the risk of bias evaluation of the included studies are shown in **Figure 2** (The risk of bias summary is provided in **Supplementary Figure S2**).

**Table 1:** Characteristics of 33 studies included in the meta-analysis.

Study	Country	Age T/C	Total T/C	Participants	Interventions		Duration and frequency	Scale
					T	C		
A.Amiri et al., 2023 (51)	Iran	56.1 ± 7.6/ 56.7 ± 6.9	30/ 30	Patients with preoperative anxiety in open heart surgery	VRET+TAU	iPad +TAU	/	①
E.Bani et al., 2019 (53)	Jordan	/	38/ 38	Breast cancer patients with chronic pain	VRT +Morphine	Morphine	/	①
J.Arifin et al., 2023 (52)	Indonesia	32.00 ± 7.91/ 34.47 ±10.32	15/ 15	Patients with surgical anxiety under regional anesthesia	VRT+TAU	Midazolam+TAU	Each 30 minutes session during the procedure	②
S.A.Almedhesh et al.,2022 (54)	Saudi Arabia	31.2/ 32.28	176/ 175	Pregnant women undergoing elective cesarean section with regional anesthesia	VRT+TAU	TAU	During the whole operation time and after regional anesthesia	③
A.Ebrahimian et al., 2022 (56)	Iran	/	31/ 31	Women with gestations of 37 to 41 weeks	VRT+TAU	TAU	First at 4 to 5 cm dilatation and second at 7 to 8 cm dilatation, each for at least 20 min	①
I.Chard et al., 2023 (55)	Britain	32 ± 9.44/ 39 ±16.86	13/ 12	Individuals diagnosed with stuttering	VRET	Waitlist	/	④
S.K.Çakir et al., 2021 (66)	Türkiye	56.33 ±11.81/ 56.20 ±15.62	30/ 30	Patients undergoing colonoscopy	VRT+TAU	TAU	5-12 minutes during the colonoscopy	①
F.E.Juarez et al., 2023 (57)	Spain	31.10 ± 4.52/ 31.60 ± 5.16	125/ 114	Women with full-termpregnancy (≥37 weeks’ gestation)	VRT+TAU	TAU	One 20-min intervention was performed during the NST and the first stage of labor	①
A.Fonseca et al., 2024 (59)	United States	46.7 ±11.3/ 41.5 ± 9.7	14/ 12	Patients undergoing surgical procedures	VRT+TAU	TAU	6 minutes during the procedure	①
A. Ghobadi et al., 2024 (61)	Iran	45.24 ±13.03/ 44.58 ±13.10	37/ 36	Patients undergoing dental implant surgery	VRT+TAU	TAU	25 minutes during the procedure	①
B.Fehlmann et al., 2023 (58)	Switzerland	45.24 ± 13.03/ 44.58 ± 13.10	43/ 46	Individuals with public speaking anxiety	VRET	Perform improvised speeches	The first session consisted of three scenes, each lasting 20 min, and the second session consisted of nine scenes, each lasting 20 min	⑤
C.Ketsuwan et al., 2022 (65)	Thailand	64.4 ± 12.2/ 65.4 ± 11.2	135/ 135	Patients undergoing office−based flexible cystoscopy	VRT+TAU	TAU	During the flexible cystoscopy	①
D.Karaman et al., 2021 (64)	Türkiye	46.9 ± 11.2/ 41.4 ± 12.9	30/ 30	Patients undergoing breast biopsy	VRT+TAU	TAU	5-6 minutes during the breast biopsy	①
E.Gokce et al., 2023 (62)	Türkiye	59.4 ± 12.1/ 58.2 ± 12.0	51/ 51	Patients undergoing coronary angiography catheter extraction	VRT+TAU	TAU	30 minutes during the procedure	①
J.G.Gonzalez et al., 2024 (60)	Spain	30.99 ± 5.01/ 30.37 ± 5.07	146/ 140	Women with full-termpregnancy (≥37 weeks’ gestation)	VRT+TAU	TAU	VR sessions were conducted before and after NST, each lasting 20 minutes	①
T.M.Hendricks et al., 2020 (63)	United States	/	10/ 10	Patients undergoing first-time sternotomy	VRGT	Non-VR tablet based game	Before procedure, the duration last 20 minutes	①
M.Keshvari et al., 2021 (67)	Iran	50.95 ± 4.12/ 52.08 ± 4.00	40/ 40	Patients undergoing coronary angiography	VRT	Placebo	Before procedure, the duration last 5 minutes	①
G.Kurt et al., 2024 (70)	Türkiye	30.33 ± 6.03/ 30.66 ± 5.75	64/ 64	Women undergoing pelvic examination	VRT+TAU	TAU	5-7 minutes during the examination	①
I.Kleiner et al., 2024 (68)	Israel	29.3 ± 4.8/ 30.3 ± 5.5	66/ 66	Women undergoing extra-amniotic balloon insertion	VRT+TAU	TAU	During the extra-amniotic balloon insertion	①
S.Y.Ko et al., 2024 (69)	China	41.13 ± 11.69/ 47.08 ± 14.15	40/ 40	Patients undergoing wound-closure procedures	VRT+TAU	TAU	During the wound-closure procedures	⑧
I.M.A.Reinders et al.,2022 (72)	Netherlands	39.9 ± 10.5/ 40.6 ± 7.8	40/ 43	Women suffering from abnormal uterine bleeding	VRET+TAU	TAU	Before procedure, the duration last 5 min and 17 s	①
T. Oz et al., 2024 (126)	Türkiye	36.3 ± 7.45/ 38.4 ± 9.27	50/ 50	Women undergoing outpatient gynecological surgical procedures	VRT+TAU	TAU	During the outpatient gynecological surgical procedures	①
V.G.Prabhu et al., 2024 (79)	United States	36.3 ± 7.45/ 38.4 ± 9.27	31/ 29	Women undergoing US-Guided Breast Biopsies	VRT+TAU	TAU	During the procedure	①
G.B.Turan et al., 2024 (76)	Türkiye	70.00 ± 9.82/ 65.20 ± 13.57	35/ 35	Patients undergoing coronary angiography	VRT+TAU	TAU	30-45 minutes during the procedure	⑥
G.Sahin et al., 2020 (78)	Türkiye	34.19 ± 12.01/ 36.09 ± 13.74	31/ 31	Patients undergoing a knee arthroscopy operation	VRT+TAU	TAU	55 minutes during the procedure	①
S.Rutkowski et al., 2022 (73)	Poland	/	16/ 16	COVID-19 Patients undergoing a pulmonary rehabilitation program	VRT+TAU	TAU	Five sessions per week for 3 weeks	⑦
A.Z.Turan et al., 2020 (75)	Türkiye	44.5 ± 24/ 41 ± 32	50/ 47	Patients undergoing surgery under spinal anesthesia	VRT+TAU	TAU	During the procedures	①
J.Yang et al., 2024 (77)	South Korea	57.5 ± 8.0/ 59.7 ± 7.3	44/ 44	Patients undergoing hepatocellular carcinoma patient scheduled for liver resection	VRET+TAU	TAU	Before procedure, the duration last 8 min and 34 s	⑨
R.L.Toraman et al., 2024 (74)	Türkiye	64.11 ± 5.34/ 64.31 ± 4.52	35/ 35	Patients undergoing ultrasonography-guided prostate biopsy	VRT+TAU	TAU	10–15 minutes during the procedure	①
N. Baltaci 2024 (80)	Türkiye	29.55 ± 6.09/ 30.11 ± 5.99	45/ 45	Women undergoing hysterosalpingography	VRT+TAU	Recording equipment +TAU	30 min and 15 min before and during the HSG procedure	①
O. Olasz 2024 (81)	Hungary	/	25/ 25	Young volunteers aged 18-30 years	VRT+MBT	iPad + MBT	20 minutes	⑩
D. Primavera 2024 (82)	Italy	47.51 ± 13.52/ 46.28 ± 13.40	39/ 25	Individuals had a clinical diagnosis of bipolar I or II disorder	VRT+MBCT	TAU	24 sessions for three months, 45 minutes each, twice a week	⑪
B. C. Schmid (83)	Australia	54.0 ± 15.4/ 60.0 ± 11.7	34/ 33	Women with gynecologic oncology who need to undergo surgery	VRT+TAU	TAU	Before procedure, the duration last 3 min and 34 s	⑫

Interventions: VRT, virtual reality therapy; VRET, virtual reality exposure therapy; VRGT, virtual reality game therapy; TAU, treatment as usual; MBCT, mindfulness-based cognitive therapy; MBT, mindfulness-based therapy.

Scales:①=The State-Trait Anxiety Inventory(SATI);②=The State-Trait Anxiety Inventory-6(SATI-6);③=A Novel Visual Facial Anxiety. Scale(NVFAS);④=Social Phobia Scale (SPS);⑤=Public Speaking Test state anxiety(PST);⑥=Anxiety Assessment Scale (AAS);⑦=The Hospital Anxiety and Depression Scale (HADS);⑧=Chinese version of State Trait Anxiety Inventory-Form Y1(CSTAI-Form Y1);⑨=Korean version of State Trait Anxiety Inventory-X (STAI-X);⑩=The State-Trait Anxiety Inventory-Y(SATI-Y);⑪=Self-Rating Anxiety Scale (SAS);⑫=Six-tier Visual Facial Anxiety Scale.

Others: NST, the nonstress test.

### 3.3 Effects of VRT on anxiety levels in patients with anxiety disorders

**Figure 3:**
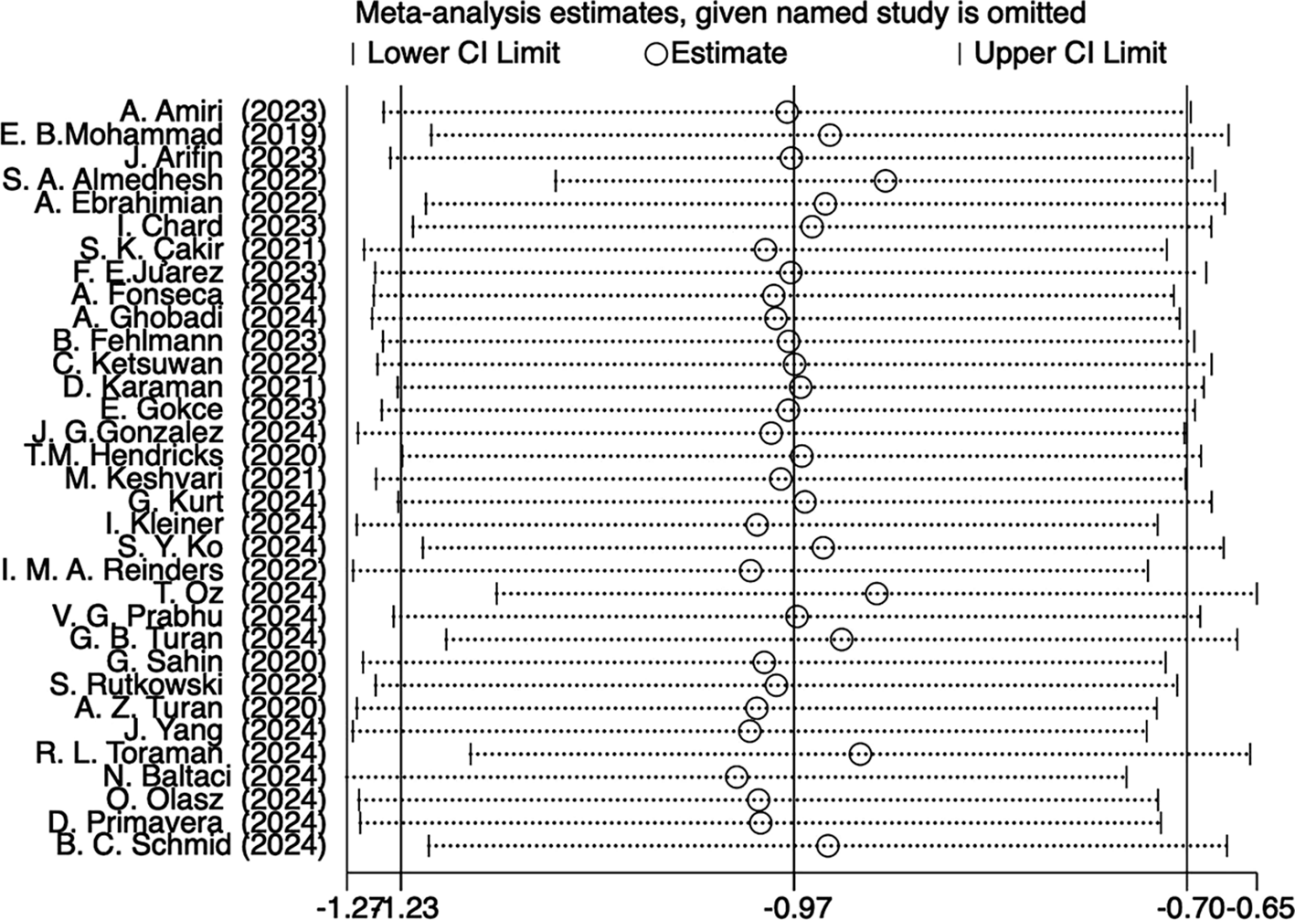
Forest plot.

**Figure 4:**
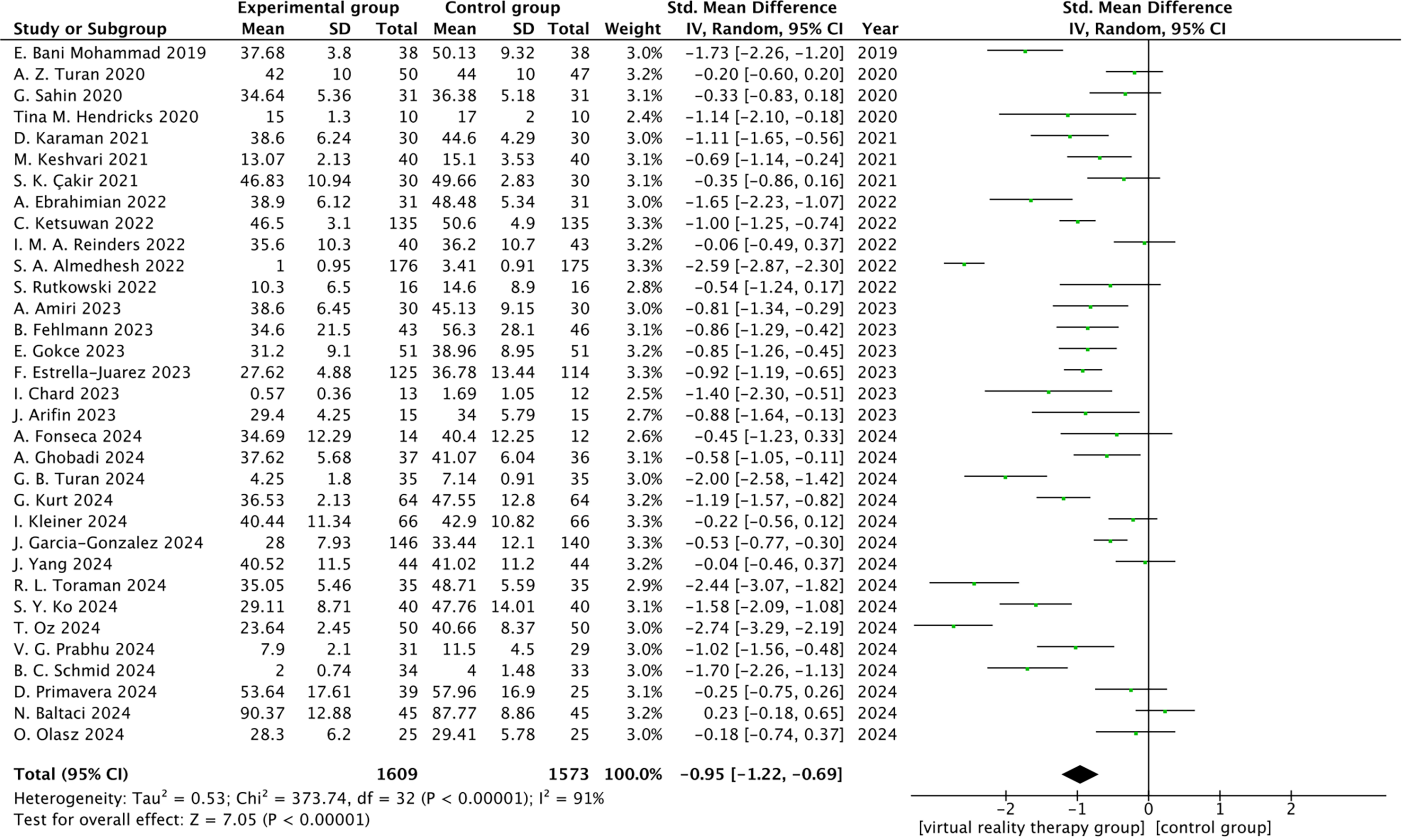
Sensitivity analysis plot.

Among The 33 studies, 26 assessed anxiety levels using the State-Trait Anxiety Inventory (SATI); 4 of these studies (52, 69, 77, 81) used different versions of the SATI, which were combined for analysis because the scoring methodology was the same; and 7 studies assessed anxiety levels using other instruments. Because evaluation tools varied widely, the standardized mean difference (SMD) was selected for effect size pooling. The results demonstrated that the analysis were statistically significant[SMD = -0.95, 95%CI (-1.22, -0.69), Z = 7.05, P < 0.00001], but there was significant heterogeneity among the studies (I^2^ = 91%, P < 0.00001) as shown in **Figure 3**. As illustrated in **Figure 4**, the results of the sensitivity analysis revealed that 11 studies (53, 54, 68, 71, 72, 74–77, 80, 83) significantly influenced the outcomes, while the remaining studies had minimal effects. Because of variations in sample numbers, virtual reality tools and techniques, assessment timing, intervention durations, and anxiety measures utilized in each study, we discovered an additional source of heterogeneity after taking into account all of the aforementioned variations. The heterogeneity was much decreased after these 2 studies(56 (69), were eliminated(I^2^ = 44%, P=0.02).According to the analysis’s findings, the difference was statistically significant [SMD = -0.76, 95%CI (-0.86,-0.67), Z = 15.59, P < 0.00001], suggesting that VRT could lower anxiety levels in patients with anxiety disorders when compared to the control group (As shown in **Supplementary Figure S3**).

### 3.4 Subgroup analyses

Subgroup analysis was carried out based on the patients’ age, gender, anxiety type, and scale, their place of origin, as well as intervention type for the Experimental group and control group. All of these factors affected the virtual reality treatment intervention effect, and the South American intervention effect was the most significant, according to the results of the subgroup analysis. The source of heterogeneity, however, is unclear and could be related to the various virtual reality devices used in the intervention, the duration of the intervention, the videos viewed by the devices, the sample sizes, and whether or not other therapies were combined. The comprehensive findings are displayed in **Table 2**.

**Table 2:** Effects of virtual reality therapy on anxiety disorders subgroup analysis.

Subgroup	Entries	Study(n)	Heterogeneity test results		Effect model	Meta-analysis results			*P* values across subgroups
			I^2^	*P*		Z	SMD (95CI%)	*P*	
Sex	Male	20	80%	<0.00001	Random	6.17	-0.81[-1.06,-0.55]	<0.00001	<0.00001
	Female	33	91%	<0.00001	Random	7.05	-0.95[-1.22,-0.69]	<0.00001	
Age	Adolescents	12	96%	<0.00001	Random	3.84	-0.95[-1.48,-0.42]	0.0005	<0.00001
	Middle age	11	74%	<0.00001	Random	5.07	-0.79[-1.09,-0.48]	<0.00001	
	Young old	5	92%	<0.00001	Random	3.54	-1.23[-1.92,-0.55]	0.0004	
Scale	SATI	26	87%	<0.00001	Random	6.83	-0.84[-1.09,-0.60]	<0.00001	<0.00001
	Other scales	7	94%	<0.00001	Random	3.44	-1.34[-2.11,-0.58]	0.0006	
Country	Asian	21	94%	<0.00001	Random	5.54	-1.08[-1.46,-0.69]	<0.00001	<0.00001
	Europe	8	66%	0.005	Random	4.19	-0.56[-0.82,-0.30]	<0.0001	
	North America	3	0%	0.43	Fixed	4.33	-0.89[-1.29,-0.49]	<0.0001	
Diagnostic	Perioperative anxiety	21	93%	<0.00001	Random	5.26	-1.05[-1.44,-0.66]	<0.00001	<0.00001
	Perinatal anxiety	3	86%	0.001	Random	3.83	-0.96[-1.46,-0.47]	0.0001	
	Examination related anxiety	3	72%	0.03	Random	4.34	-0.89[-1.29,-0.49]	<0.0001	
	Other related anxiety	6	60%	0.03	Random	2.62	-0.48[-0.84,-0.12]	0.009	
Control interventions	Active control	6	77%	0.0006	Random	2.00	-0.50[-0.98,-0.01]	0.005	<0.00001
	Inactive control	27	92%	<0.00001	Random	6.75	-1.02[-1.31,-0.72]	<0.00001	
Experimental interventions	Virtual reality distraction therapy	28	73%	0.005	Random	1.90	-0.42[-0.86,0.01]	0.06	<0.00001
	Virtual reality exposure therapy	5	92%	<0.00001	Random	6.79	-1.02[-1.31,-0.72]	<0.00001	

### 3.5 Publication bias

**Figure 5:**
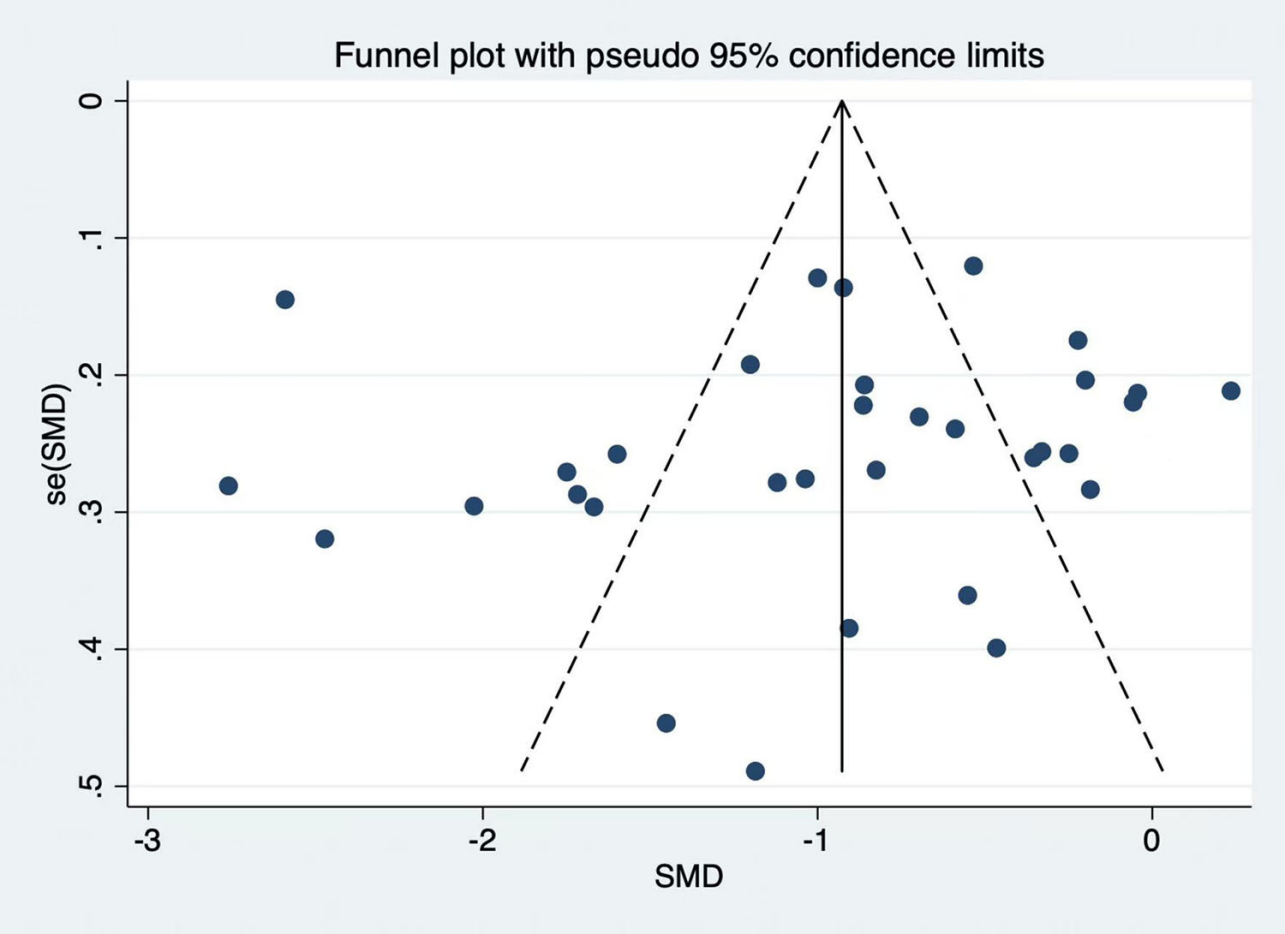
Funnel plot of publication bias.

The findings of the publication bias test, which was applied to the 33 publications in this study, indicated that there might be publication bias because the funnel plot was somewhat asymmetrical, as shown in **Figure 5**. Nevertheless, there was no discernible publication bias according to Egger’s test (P = 0.731).

## 4 Discussion

This meta-analysis evaluated virtual reality therapy’s (VRT) effectiveness in treating anxiety disorders. The criteria for inclusion were met by 33 studies, all of which were randomized controlled trials. Of these, 27 studies included an inactive control group that received usual treatment (i.e., participants received usual medication, health education, and usual care), waitlist, or placebo. Six studies’ control group included active intervention, which included presenting the experimenter with a public speech, utilizing an iPad for preoperative health education, using a tablet device to perform mindfulness exercises, listening to nature sounds using a recording device, and using a non-VR video game. This study examined the impact of virtual reality treatment on individuals with anxiety disorders and came to the conclusion that virtual reality-based interventions can lessen anxiety and help patients with anxiety disorders feel better[SMD = -0.95, 95%CI (-1.22,-0.69), Z = 7.05, P < 0.00001]. Simultaneously, the meta-analysis expanded the VR-based interventions, limited the participants’ condition kinds, and became more focused. Of the 33 included studies, participants’ anxiety types included perioperative anxiety in 21 studies, perinatal anxiety in 3 studies, anxiety during a medical examination in 3 studies, anxiety related to their own illness in 2 studies, public speaking anxiety in 1 study, bipolar disorder related anxiety in 1 study and social anxiety in 2 study. In addition, all 33 eligible studies were published within the last 5 years, which may reflect the development of virtual reality technology and its application in the field of mental health as a research hotspot in recent years. Notably, there is conflicting evidence about VR’s ability to reduce perioperative anxiety. Some studies have shown that VR therapy does not reduce perioperative anxiety (84–86), while others have shown some positive effects (87–91). Three previous studies have shown that virtual reality therapy has been used in patients before surgery with significant results, especially in children (90–92). The inconsistency in the results of these studies may be due to the use of different anxiety scales. The most widely used measure for assessing perioperative anxiety is the Spielberger State and Trait Anxiety Inventory (STAI). Some more focused measures are the Yale Preoperative Anxiety Scale (93), the Amsterdam Perioperative Anxiety and Information Scale (94), the State-Trait Operation Anxiety (95), and others. These scales differ in terms of item content, questionnaire length, and scoring standards, all of which will affect the study’s findings. Therefore, when assessing the effectiveness of VR intervention on perioperative anxiety, it is necessary to take into account the usage of various scales.

Because mental health issues are inextricably linked to their surroundings, individuals can use an interactive virtual reality environment to see whether treatment is feasible. The treatment of mental illnesses has made extensive use of virtual reality in recent years. Distraction therapy and exposure therapy are among the therapeutic modalities. Virtual reality technology plays a major role in treating anxiety disorders by creating an exposure environment. By exposing anxious individuals to frightening stimuli or scenes, virtual reality exposure treatment might help them become desensitized to and eventually eradicate their fear. It has been demonstrated that this method works well for treating some phobias, including panic disorder (PD), social anxiety disorder (SAD), post-traumatic stress disorder (PTSD), and generalized anxiety disorder (GAD) (96).Conventional exposure therapy requires the presentation of real-world stimuli; however, certain stimuli, like driving, flying, altitude, etc., are extremely risky and unlikely to occur in the actual world. Furthermore, it is not practical to use typical exposure therapy for postwar trauma. As a result, virtual reality exposure therapy can effectively compensate for the limited circumstances and incomplete treatment of traditional exposure therapy. In contrast to traditional imagined exposure therapy, virtual reality exposure therapy, according to Emmelkamp et al. (97), can help patients visualize stimuli and environments that they are unable to accept by simulating real experiences and offering personalized and unique scenes for exposure therapy. Park et al. (98) think that virtual reality technology can give psychotherapists a safe, controlled, and controllable environment, increasing the viability and efficacy of exposure treatment. In order to help patients who are afraid of heights learn to overcome their phobia, Hong et al. (99) employed virtual reality to imitate high altitude. Zainal (100), Premkumar (101), Rubin (102) and others used VRET to help people with social anxiety overcome speech anxiety, and the treatment effect was relatively stable after 4 to 6 years of follow-up. The majority of research on VRET’s application in treating PTSD has been on war, auto accidents, and terrorist incidents (103–105). Lehoux et al. (106) used virtual reality to treat substance use disorders (SUD). Additionally, virtual reality was employed by Riva et al. and Corno et al. (107, 108) to treat people with eating disorders. The treatment of obsessive-compulsive disorder has also benefited from advancements in virtual reality technology. Javaherirenani et al. (109) verified that treating patients with clean pollution-induced obsessive-compulsive disorder with virtual exposure therapy is feasible.

According to Stanney et al. (110), over 80% of VR users encounter some related negative effects, despite the fact that the advantages of VR have been extensively documented in the literature. Potential negative health impacts of VR exposure have also been noted in a number of earlier research (111, 112). Health issues include adverse symptoms include nausea and vertigo, confusion, and exhaustion of the muscles (113). Meanwhile, extended usage of VR devices can raise the risk of nearsightedness or hyperopia and cause eye strain, dryness, and impaired vision (114). Macular degeneration is more likely to occur if blue light from VR device screens damages retinal cells over time (115). Long-term VR immersion can also result in psychological issues including addiction, social anxiety, depression, and phobias, as well as physical issues like brain fatigue, neurasthenopia, poor focus, and memory loss (116). Aside from potential health risks, VR-related side effects may also detract from the user experience.

In distraction intervention therapy, virtual reality takes advantage of the features of its 3D reality environment to draw patients’ attention away from the things that make them anxious or in pain in order to alleviate such symptoms. Using VR virtual reality equipment, the intervention content of distraction therapy is different. For example, children’s puncture process can be used to divert their attention, lower their anxiety and fear, and significantly increase the puncture success rate by using VR equipment to enjoy music, movies, animations, games, or exposure to the beach and other natural environments (117–119). Apart from its use in children, virtual reality-based distraction therapy has also been applied to adult cancer patients in hospitals to manage anxiety (120–122). In Bani et al.’s study (121), for instance, hospitalized patients with breast cancer during chemotherapy were assisted using VR virtual reality equipment. Current anxiety can be reduced by diverting the patient’s focus from the stressor’s acute stimulus. It has not been proven whether distraction therapy’s ability to reduce anxiety has a long-lasting effect, and the patient’s anxiety may return once they are back in the real world without the interactive environmental stimulation that VR technology offers. Thus, In order to continually alleviate patients’ anxiety symptoms, enhance their degree of adaptability, and encourage them to adjust as quickly as possible to subsequent stresses, it is necessary to improve the intervention content of VR equipment. Since VR virtual reality equipment can create a simulated three-dimensional environment, patients can be temporarily removed from stressful situations. The 3D scene that the equipment provides can also serve as a setting for mindfulness meditation, allowing patients to focus on mindfulness meditation exercises and improving their cognitive abilities to deal with illnesses or negative emotions. Adapt to stressors as quickly as possible. The effectiveness of mindfulness meditation interventions has been demonstrated to be improved by VR technology. For example, in a study by Lee et al. (123), critically ill patients’ sleep quality was enhanced by using VR virtual reality technology in conjunction with mindfulness meditation, increased the mindfulness meditation intervention’s impact significantly. Nevertheless, VR-based mindfulness research in non-clinical populations is still in its early stages, and future advancements in experimental research design, psychometric tools and indicators, and technical equipment operation are required (124).

In conclusion, the use of virtual reality technology in treatment is growing in popularity and affordability as a result of technological advancements (125). Virtual reality technology, on the other hand, has demonstrated great potential in the assessment and management of anxiety disorders and compensates for many of the drawbacks of conventional approaches. Nonetheless, there are a few things that the current study has to take into account. First and foremost is the patient’s sense of presence. The sense of presence is not only an important metric for analyzing virtual reality technology, but it also plays a significant role in generating dread and panic and finally achieving the intended therapeutic effect. However, few studies have considered the impact of context on the outcome of assessment and treatment. Second, the research should take into account any potential negative effects that virtual reality technology may have on some patients, such as nausea and vertigo.

The majority of current research is still in the preliminary stages of investigation, and more research is required to determine whether virtual reality may be used effectively in the future to diagnose and treat anxiety disorders. In terms of evaluation, the first step is to standardize the VR evaluation procedure in order to establish a comparatively fixed process or model and to elucidate its validity and reliability. Second, more physiological indicators, like heart rate, blood pressure and brain nerve activity, can be combined in the future to examine the relationship between cognitive, behavioral, and physiological indicators because VR can be compatible with other technologies, such as eye movement, electroencephalography (EEG), and brain imaging technologies. Regarding treatment, in order to further validate the effectiveness of VRT on anxiety disorders, a bigger sample size, the inclusion of various patient subtypes, a more rigorous experimental design conducted under controlled conditions, and the combination of pertinent physiological indicators are required; Second, additional research is required to examine the viability and efficacy of VRT in clinical settings. Third, long-term follow-up data are required to observe the effect of VRT on patient treatment. Fourth, in order to properly direct the design of virtual reality, additional research is required to understand the causal relationship between the sense of presence and anxiety, as this is a significant factor influencing patients’ worry and terror. Fifth, VRT still requires the participation of therapists, which means limited treatment duration and high significant treatment expenses. In the future, the participation of therapists and the threshold of treatment can be reduced (such as app and recorded instructions), so that more people can receive evidence-based psychotherapy. Furthermore, considering the possible negative effects of virtual reality, the duration of use can be appropriately controlled, and the device can be removed in time to rest after dizziness and other discomfort symptoms occur. From the perspective of VR designers, future products can be equipped with chips with stronger computing power to reduce delay and reduce the sense of vertigo when using VR equipment as much as possible. It can also make VR equipment lighter and more ergonomic to further improve user wearing comfort. In addition, user health systems can be developed to monitor user experience in real time. Finally, to further investigate the therapeutic effects of VRT, future studies could concentrate on analyzing and measuring the impact of VR devices alone on mental health as opposed to in conjunction with other psychotherapy techniques.

## 5 Conclusions

According to available data, VRT is a successful treatment for anxiety disorders. It also lessens the symptoms of anxiety disorders, albeit it is not a complete improvement over traditional treatment. The number and caliber of the included studies place restrictions on the aforementioned research findings. More focused, large-sample, high-quality research on many kinds of anxiety disorders is required in the future for verification.

## Data Availability

The original contributions presented in the study are included in the article/**Supplementary Material**. Further inquiries can be directed to the corresponding author.

## Supplementary material

Supplementary Figure 2 Risk of bias summary.

Supplementary Figure 3 Forest plot after sensitivity analysis.
