# Supplementary figures and images for: Effectiveness of virtual reality therapy in the treatment of anxiety disorders in adolescents and adults: a systematic review and meta-analysis of randomized controlled trials
Source: Front Psychiatry. 2025 Feb 27;16:1553290. doi: 10.3389/fpsyt.2025.1553290 (PMC11904249; doi:10.3389/fpsyt.2025.1553290)

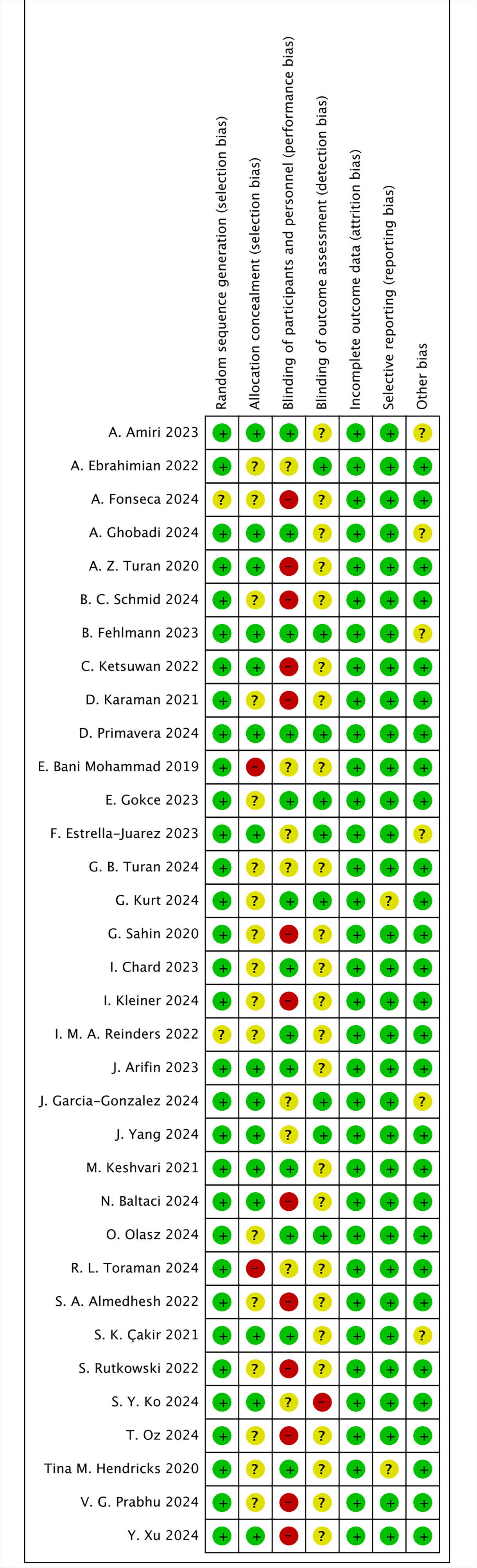

Supplement: Supplementary Figure 2 — Risk of bias summary. [file Image1.tif]

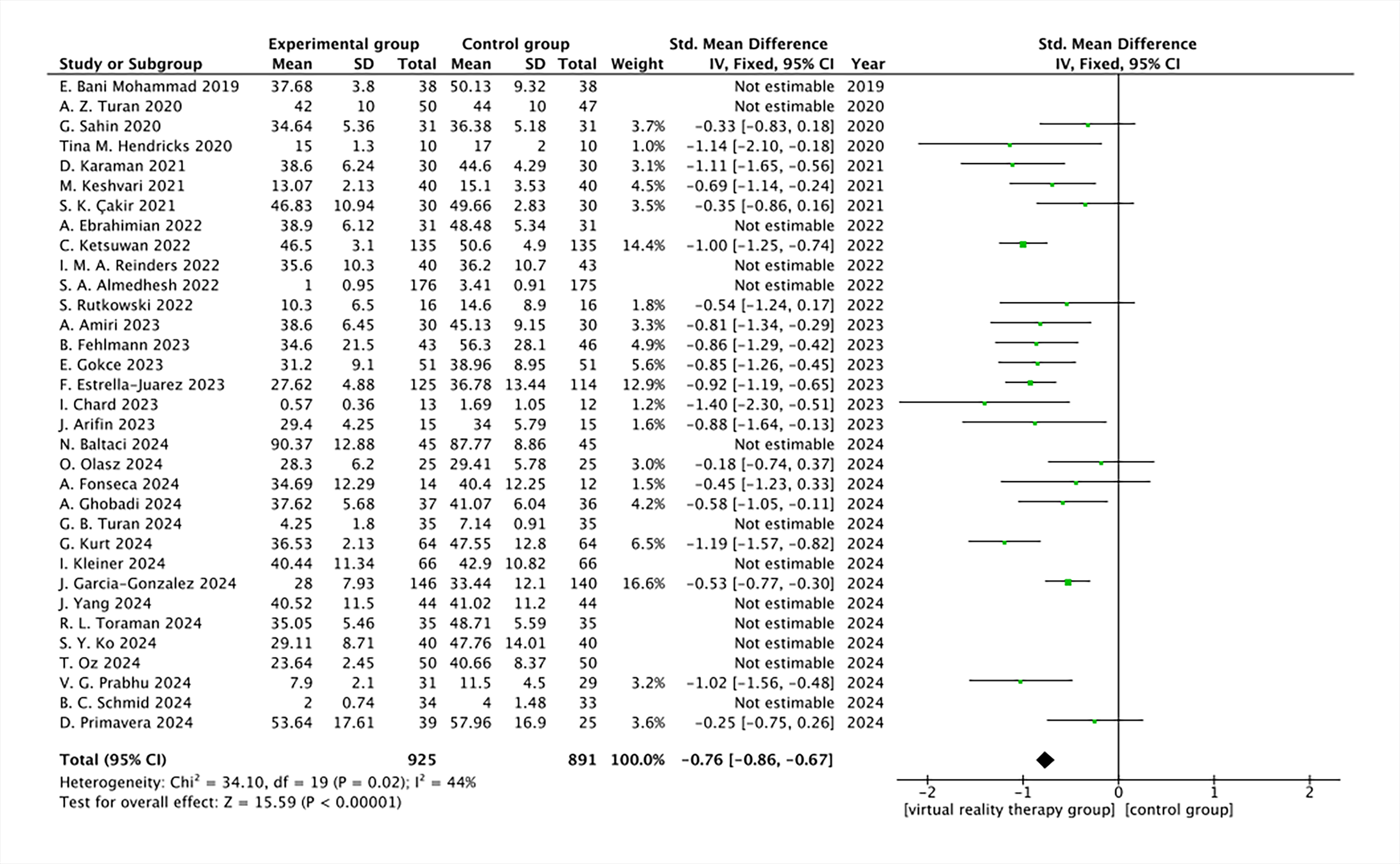

Supplement: Supplementary Figure 3 — Forest plot after sensitivity analysis. [file Image2.tif]
