# Supplementary material for: Effectiveness of virtual reality therapy in the treatment of anxiety disorders in adolescents and adults: a systematic review and meta-analysis of randomized controlled trials
Source: Front Psychiatry. 2025 Feb 27;16:1553290. doi: 10.3389/fpsyt.2025.1553290 (PMC11904249; doi:10.3389/fpsyt.2025.1553290)
Supplement: Supplementary file 3 [file Table1.docx]

Table S1

Search strategy used in PubMed database

| Number | Search terms |
| --- | --- |
| #1 | “anxiety”[Title/Abstract]OR“angst”[Title/Abstract]OR“nervousness”[Title/Abstract]OR“hypervigilance”[Title/Abstract]OR“anxiousness”[Title/Abstract]OR“anxiety disorder”[Title/Abstract]OR“anxiety neuroses”[Title/Abstract]OR“neurotic anxiety state”[Title/Abstract] |
| #2 | “adolescent”[Title/Abstract]OR“youth”[Title/Abstract]OR“young”[Title/Abstract]OR“teen”[Title/Abstract]OR“teenager”[Title/Abstract]OR“teenage”[Title/Abstract]OR“young adult”[Title/Abstract]OR“adult”[Title/Abstract] |
| #3 | “VR”[Title/Abstract]OR“virtual reality exposure therapy”[Title/Abstract]OR“virtual reality immersion therapy”[Title/Abstract]OR“virtual reality therapy”[Title/Abstract]OR“virtual reality”[Title/Abstract]OR“augmented reality”[Title/Abstract]OR“artificial reality”[Title/Abstract]OR“computer-simulated reality”[Title/Abstract]OR“computer-simulated environment”[Title/Abstract]OR“mediated reality”[Title/Abstract] |
| #4 | #1 AND #2 AND #3 |
